# Wild emmer wheat, the progenitor of modern bread wheat, exhibits great diversity in the *VERNALIZATION1* gene

**DOI:** 10.3389/fpls.2022.1106164

**Published:** 2023-01-06

**Authors:** Beáta Strejčková, Elisabetta Mazzucotelli, Radim Čegan, Zbyněk Milec, Jan Brus, Esra Çakır, Anna Maria Mastrangelo, Hakan Özkan, Jan Šafář

**Affiliations:** ^1^ Institute of Experimental Botany of the Czech Academy of Sciences, Centre of the Region Haná for Biotechnological and Agricultural Research, Olomouc, Czechia; ^2^ Department of Cell Biology and Genetics, Faculty of Science, Palacký University Olomouc, Olomouc, Czechia; ^3^ Council for Agricultural Research and Economics (CREA) Research Centre for Genomics and Bioinformatics via San Protaso 302, Fiorenzuola d’Arda, Italy; ^4^ Department of Plant Developmental Genetics, Institute of Biophysics of the Czech Academy of Sciences, Brno, ;Czechia; ^5^ Department of Geoinformatics, Faculty of Science, Palacký University Olomouc, Olomouc, Czechia; ^6^ Department of Field Crops, Faculty of Agriculture, University of Çukurova, Adana, Turkey; ^7^ Council for Agricultural Research and Economics (CREA) Research Centre for Cereal and Industrial Crops, Foggia, Italy

**Keywords:** *VERNALIZATION1*, next generation sequencing, heading time, GWAS, wild emmer wheat

## Abstract

Wild emmer wheat is an excellent reservoir of genetic variability that can be utilized to improve cultivated wheat to address the challenges of the expanding world population and climate change. Bearing this in mind, we have collected a panel of 263 wild emmer wheat (WEW) genotypes across the Fertile Crescent. The genotypes were grown in different locations and phenotyped for heading date. Genome-wide association mapping (GWAS) was carried out, and 16 SNPs were associated with the heading date. As the flowering time is controlled by photoperiod and vernalization, we sequenced the *VRN1* gene, the most important of the vernalization response genes, to discover new alleles. Unlike most earlier attempts, which characterized known *VRN1* alleles according to a partial promoter or intron sequences, we obtained full-length sequences of *VRN-A1* and *VRN-B1* genes in a panel of 95 wild emmer wheat from the Fertile Crescent and uncovered a significant sequence variation. Phylogenetic analysis of *VRN-A1* and *VRN-B1* haplotypes revealed their evolutionary relationships and geographic distribution in the Fertile Crescent region. The newly described alleles represent an attractive resource for durum and bread wheat improvement programs.

## 1 Introduction

Bread wheat (*Triticum aestivum* L.) and durum wheat (*T. turgidum* L. var. *durum* Desf.) are essential crops. Wheat has been the staple food of the major civilizations of Europe, West Asia, and North Africa for 8 000 years. The origin of these species is closely related to the development of human civilization. Although bread wheat is most successful between the latitudes of 30° and 60°N and 27° and 40°S (Nuttonson, 1955), durum wheat is grown mainly in the Mediterranean area (Royo et al., 2017). These species’ spread from their site of origin is connected with the ability to adapt to various climates and time their flowering to favorable conditions (Dubcovsky and Dvorak, 2007). This is controlled by the flowering pathway, consisting of photoperiod and vernalization pathways. While the photoperiodic pathway promotes flowering under long-day conditions, the vernalization pathway is induced by cold. Major vernalization genes are *VERNALIZATION1* (*VRN1*), *VERNALIZATION2* (*VRN2*), and *VERNALIZATION3* (*VRN3*). *VRN1*, coding for a MADS-box type II transcription factor homologous to *Arabidopsis APETALA1* (Yan et al., 2003), acts as a floral activator and is expressed in leaves and shoot apical meristem (Jin and Wei, 2016). VRN1 protein has a typical “MIKC” structure, formed by four domains: MADS (DNA binding), I (intervening; for dimerization), K (keratin-like; involved in dimer and tetramer formation), and C-terminal (carboxyl-terminal; variable, important for transactivation and higher-order complex formation) (Egea-Cortines et al., 1999; Saedler et al., 2001; Becker and Theißen, 2003; Lai et al., 2021). In wheat with winter growth habit, *VRN1* alleles are intact, and their expression is induced by a period of cold treatment called vernalization (Trevaskis et al., 2003). Natural mutations of *VRN1* promoter or intron 1 regions cause strong expression of the gene resulting in spring growth habit (Fu et al., 2005). In general, intron 1 mutations causing the spring growth habit span so-called ‘critical region’, which seems to comprise important regulatory site(s) (Fu et al., 2005), such as the RIP3 region, a binding site of the putative flowering repressor TaGRP2 (Xiao et al., 2014). It was shown that sequence variability of recessive *vrn-A1* allele can affect its basal expression and the vernalization requirement. Hexaploid cultivar Jagger with the *vrn-A1a* allele requires three weeks of vernalization treatment whereas cultivar 2174 carrying the *vrn-A1b* allele containing exon 7 mutation needs six weeks (Li et al., 2013).

Wild emmer (*T. turgidum* ssp. *dicoccoides*) is an allotetraploid (2n=4x=28; genomes BBAA) species. Genome B was derived from an ancient species closely related to the extant *Aegilops speltoides*, while genome A was derived from *T. urartu* (Mayer et al., 2014). This wild wheat is easily crossed with durum and common wheat, yielding F1 hybrids, which are fully or partially fertile (Harlan and Wet, 1971). Pairing between the “wild” and “domesticated” chromosomes of the B and A genome is complete. Thus, almost all alleles can be transferred by simple plant breeding procedures from the “wild” into their “domesticated” homologues. Wild emmer is native to the Fertile Crescent, where it grows in two main regions: the Syria-Israeli and the Turkish-Iranian, where it occupies a variety of primary and secondary habitats (Maccaferri et al., 2019). Wild emmer wheat harbors rich allelic diversity for numerous important traits, including agronomic characteristics, grain quality, and resistance to biotic and abiotic stresses (Nevo et al., 2012; Fadida-Myers et al., 2022). A large number of genes and QTLs that are valuable for wheat improvement have been identified in the wild emmer gene pool and mapped (Reader and Miller, 1991; Peng et al., 2000; Rong et al., 2000; Chee et al., 2001; Peng et al., 2003; Merchuk-Ovnat et al., 2016; Balla et al., 2022a; Balla et al., 2022b).

Here, we present the heading time data for a panel of 263 wild emmer wheat genotypes collected in the Fertile Crescent and GWAS analysis of 178 winter genotypes from the panel. We have selected 95 genotypes with diverse growth habits and flowering times and sequenced their full *VRN-A1* and *VRN-B1* genes. We discovered several mutations in both coding and non-coding regions and examined the expression of seven selected genotypes. Identifying novel alleles causing early/late flowering could bring value to the breeding of improved genotypes.

## 2 Materials and methods

### 2.1 Plant material and growth conditions

#### 2.1.1 Phenotypic evaluation in the field

A total number of 263 wild emmer wheat genotypes (*T. dicoccoides*; **Supplementary Table 1**) from the region of Fertile Crescent, including both spring and winter growth types (according to the GRIN-Global database (Version: 2.0.5.0) (https://npgsweb.ars-grin.gov/gringlobal/search)) was used for heading date experiments in Turkey (2016/2017, 2017/2018 and 2018/2019; Adana (37.015962, 35.355740)) and Italy (2016/2017; Fiorenzuola d’Arda (44.9276601, 9.8945892), 2017/2018 and 2018/2019; Foggia (41.460391, 15.501311)). In addition growth habit was retrieved for most of the accessions from passport data provided by Genebanks. First, seeds were germinated in pots under controlled conditions in the greenhouse (long day, 16 h of light at 20°C and 8 h of darkness at 16°C). At the third-leaf stage, seedlings were transferred to the field and planted in the 1m row (seven plants from the same genotype in one row), which was done in two replicates for each genotype. When the spike emerged in 75% of the plants in the row, the day was fixed as the heading date.

#### 2.1.2 DNA extraction for VRN1 sequencing

We selected 95 wild emmer wheat genotypes to cover early to late flowering genotypes (**Supplementary Table 2**). *T. dicoccoides* line Tabigha 15 for sequencing of the *Vrn-A1d* allele was provided by The Institute of Evolution Wild Cereal Gene Bank at the University of Haifa. Seeds were imbibed on Petri dishes at 21°C for 48 h and then placed at 4°C for two days to synchronize germination. Seedlings were transferred to a pot and placed in the growth room under long-day conditions (16 h of light at 20°C and 8 h of darkness at 16°C). For DNA isolation, leaves were collected three weeks after potting.

### 2.2 SNP genotyping

The DNA was extracted from 8–10 days-old seedlings (4-5 plants pooled) following the CTAB method. After standard Axiom sample preparation, all accessions were genotyped with the 35 K Wheat Breeders Affymetrix-SNP array (Axiom Wheat Breeder’s Genotyping Array). Allele calling was carried out using the Affymetrix proprietary software package GTC, following the Axiom^®^ Best Practices Genotyping Workflow. In addition, all genotypes were genotyped for the *Ppd-A1* allele, as reported in Beales et al. (2007).

### 2.3 Genome-wide association study

The Genome-Wide Association Study (GWAS) for heading time in winter genotypes was run by GAPIT version 3 using a fixed and random model circulating probability unification (FarmCPU) (Liu et al., 2016), and the Bayesian information and Linkage-disequilibrium Iteratively Nested Keyway (BLINK) model (Huang et al., 2019). The GWAS for all data obtained from different locations and years was done from BLUP values (Best Linear Unbiased Predictions) which can eliminate the environmental and year deviations. BLUP values were counted with R package Phenotype (Piepho et al., 2008). Since the SNP frequency is not too high in *T. dicoccoides* genome to perform linkage disequilibrium analysis (most of the SNPs were alone in blocks; data not shown), the candidate genes were searched 500kb upstream and downstream from the SNP position. The protein sequences of candidate genes were extracted from *T. dicoccoides* genome (https://plants.ensembl.org/Triticum_dicoccoides/Info/Index) and annotated based on the best UniProtKB/Swiss-Prot blastp hit online at https://blast.ncbi.nlm.nih.gov/. The panel of 178 winter genotypes used for GWAS, including their heading time data and BLUP values, is listed in **Supplementary Table 3**.

### 2.4 PCR amplification and sequencing

Genomic DNA for sequencing was extracted from leaves of two-week-old plants using NucleoSpin Plant II (MACHEREY-NAGEL, Germany) according to the manufacturer’s instructions.

Sequences of *VRN1* genes were obtained by sequencing overlapping PCR products using several sequencing protocols as described in (Strejčková et al., 2021). Briefly, short PCR products (< 1200 bp) were sequenced by the Sanger method, while long PCR products (> 2700 bp) were sequenced with the Illumina iSeq platform.

#### 2.4.1 PCR amplification

DNA was amplified using Bio-Rad C1000 Touch Thermal Cycler (Bio–Rad, USA). Primers and conditions used for PCR are listed in **Supplementary Table 4**.

#### 2.4.2 Sanger sequencing

PCR clean-up was performed by ExoSap (ThermoFisher Scientific, USA). The sequencing reactions were performed using the BigDye1 Terminator v3.1 Cycle Sequencing Kit (Applied Biosystems, USA) and purified using the Agencourt Clean SEQ Dye-Terminator Removal kit (Beckmann Coulter, USA). The reactions were analyzed on the ABI3730xl DNA analyzer (Applied Biosystems, USA). The resultant sequences were trimmed and assembled using Geneious Prime^®^ 2022.0.1 (http://www.geneious.com). Assemblies were verified by alignment with the reference *VRN1* sequence of Triple Dirk C (TDC) (Genbank accessions AY747600.1 and AY747604.1).

#### 2.4.3 Illumina sequencing

PCR amplicons were purified using AMPure XP Beads (Beckman Coulter, USA) with a DNA volume/beads ratio of 1:1. DNA was quantified using the Qubit dsDNA HS assay system (Invitrogen, USA). For each PCR amplicon or pool of amplicons, a sequencing library was prepared using the NEBNext^®^ Ultra™ II DNA Library Prep Kit for Illumina^®^ with the following modifications: (i) DNA was fragmented in 50 µL solution using a Bioruptor Plus (Diagenode, Belgium) eight times for 30 s on the HIGH setting; (ii) size selection was performed for an approximate insert size of 500–700 bp; and (iii) PCR enrichment was carried out in 3–4 cycles. Libraries were equimolarly pooled and sequenced on an Illumina iSeq system with 150 bp paired-end (PE) reads to achieve a minimal amplicon coverage of 100×.

### 2.5 Sequencing data analysis

Read trimming based on quality (Q30) and sequencing adaptor removal were performed with Trimmomatic (v.0.32) (Bolger et al., 2014). All trimmed reads for each sample were mapped to the *VRN1* TDC reference with BWA-MEM (v.0.7.15) (Li, 2013). Mapped reads for each genome variant (A and B) were extracted from the bam file by SAMtools (v.1.9) (Li et al., 2009) and *de novo* assembled by Spades (v.3.13.0) (Bankevich et al., 2012). Mapping results were manually reviewed with the Integrative Genome Viewer v.2.6.3 (IGV) (Robinson et al., 2017). Obtained sequences were further analyzed in Geneious Prime^®^ 2022.0.1 (http://www.geneious.com).

### 2.6 *In silico* protein analysis

VRN1 protein domains were identified using the InterPro database (Mulder et al., 2007). The secondary structure of proteins was predicted using the module EMBOSS Protein Analysis version:1.0 (Rice et al., 2000) in the Geneious Prime^®^ 2022.0.1. Protein alignments were done in the Geneious Prime^®^ 2022.0.1 using MAFFT v7.490 (Katoh et al., 2002; Katoh and Standley, 2013).

### 2.7 Phylogenetic analysis and maps

Nucleotide alignments for phylogenetic analysis were conducted in Geneious Prime^®^ 2022.0.1 (http://www.geneious.com) by MAFFT v7.490 (Katoh et al., 2002; Katoh and Standley, 2013). The evolutionary history was inferred using the Neighbor Joining tree build method (Saitou and Nei, 1987) and Tamura-Nei genetic distance method (Tamura and Nei, 1993). The bootstrap consensus tree is inferred from 1000 replicates. Data preparation for map-making was done using Microsoft Excel. The maps were created using ArcGIS Pro 3.0.2 https://www.esri.com/en-us/arcgis/products/arcgis-pro/resources.

### 2.8 RNA extraction and gene expression analysis

We selected seven genotypes (PI 538660, PI 538646, PI 466935, PI 471057, PI 466991, PI 428082, and PI 487263) with mutated alleles and the hexaploid cultivar Triple Dirk C (TDC) to analyze *VRN-1* transcription. Seeds were imbibed on Petri dishes and placed at 4°C for three days to synchronize germination. Six seedlings of each variety were transferred to pots and placed in a growth chamber under LD conditions for two weeks). Afterward, they were vernalized for four weeks (8 h of light at 6°C and 16 h of darkness at 6°C). Finally, they were returned to the growth chamber under LD conditions. We collected leaves two, four, six, and eight weeks after potting. Total RNA was extracted using a *Quick*-RNA Miniprep Kit (Zymo Research, USA), including DNAse treatment according to the manufacturer’s instructions. cDNA was synthesized using a RevertAid First Strand cDNA Synthesis Kit (Thermo Scientific™, USA) according to the manufacturer’s instructions using 2 μg of total RNA and anchored-oligo (dT)_18_ primers. The gene expression level was determined using reverse transcription-qPCR (RT–qPCR). RT–qPCR was performed using qPCR 2x SYBR Master Mix (Top-Bio, Czech Republic) on the CFX96TM Real-Time PCR Detection System (Bio–Rad, USA). The data were analyzed using the 2^-ΔΔCq^ method with CFX Maestro 2.0 software (Bio–Rad, USA). Three biological replicate PCR amplifications were performed for each sample. The expression level was standardized against the reference *glyceraldehyde-3-phosphate dehydrogenase* (*GAPDH*), according to Ivaničová et al. (2017). Sequences of all primers used for RT–qPCR are listed in **Supplementary Table 5**. We also scored heading time when half of the first spike had emerged.

### 2.9 Statistical analysis

The statistical significance of expression data was determined by one-way analysis of variance (ANOVA), followed by post-hoc Duncan’s Multiple Range test (α=0,05). ANOVA was performed in Statistica 14.0.0.5 (Statsoft).

## 3 Results

### 3.1 Heading time differences in the collection of wild emmer wheat

Phenotypic data were obtained for the collection of 286 WEWs from the Fertile Crescent. All genotypes carry the photoperiod-sensitive *Ppd-A1* allele. The heading date (HD) was scored in three consecutive years in two different countries - Turkey (TUR) (**Supplementary Table 6**) and Italy (IT) (**Supplementary Table 7**). Mean heading time ranged from 124 to 156 days (TUR) and from 143 to 170 days (IT).

**Figure 1:**
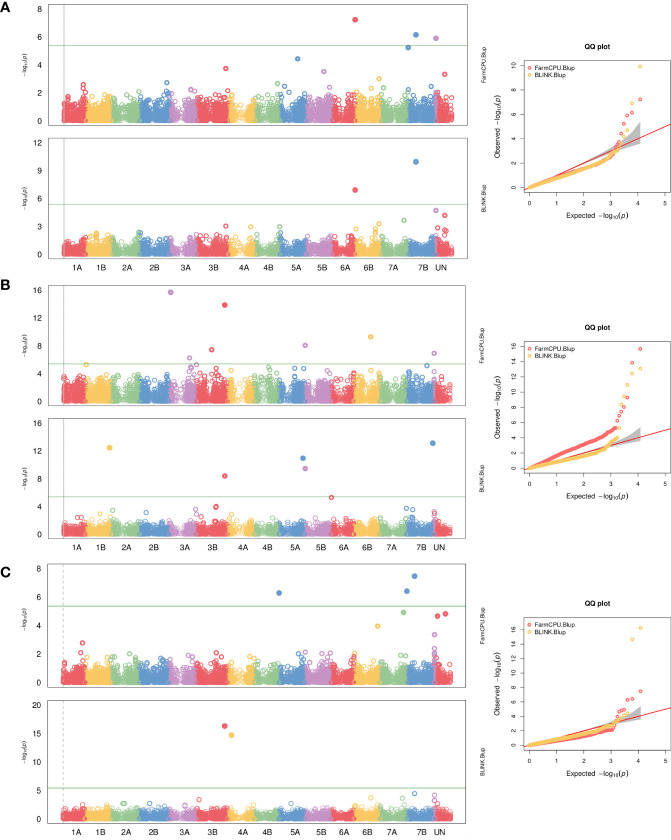
Genome-wide association study (GWAS) result plots for heading date in the wild emmer wheat population. Manhattan and Quantile-quantile plots for BLINK and FarmCPU models for data from Italy **(A)**, Turkey **(B)**, and for both localities **(C)**. The significant threshold (p=0.05) is indicated by the green horizontal line.

Within the large WEW collection evaluated for HD, a panel of 178 genotypes with winter growth habit was selected to be used in GWAS analysis based on 12,101 polymorphic SNPs from the Axiom 35K wheat breeder array. In the GWAS for heading date (density histograms for observed phenotypic data are presented in **Supplementary Figure 1**), sixteen highly significant marker-trait associations were identified by GAPIT (seven by FarmCPU and nine by BLINK model). They were located on chromosomes 1B (1), 3A (2 and 3), 3B (4 and 5), 4A (6), 5A (7 and 8), 5B (9), 6A (10), 6B (11), 7B (12, 13, and 14) and ND (not determined, two SNPs), explaining 2% to 36% of the phenotypic variations. The allelic effect for SNP markers associated with the heading date is shown in **Supplementary Figure 2**. The details of these markers are summarized in **Supplementary Table 8** and depicted as Manhattan and QQ plots in **Figure 1**. Candidate genes are listed in **Supplementary Table 9**. Out of the 153 candidate genes, 122 were annotated based on the UniProtKB/Swiss-Prot blastp hit. Some of the orthologs were reported to have a function in the flowering regulation of other species or are associated with the epigenetic regulation of plant development. None of the MTA is coincident with known *VRN* loci because of the array’s SNP coverage.

### 3.2 *VRN1* sequence variability in a subset of 95 wild emmer wheat

We selected 95 WEW from the phenotyped panel to inspect the allelic diversity of the important flowering time regulator, the *VRN1* gene, in both spring and early to late winter genotypes. Forty-one of the 95 selected genotypes are from Turkey, 29 from Israel, three from Syria, ten from Palestina, and 12 from Lebanon. The mean heading time of these genotypes ranges from 124 to 156 days when grown in Turkey and 143 to 170 days in Italy.

**Figure 2:**
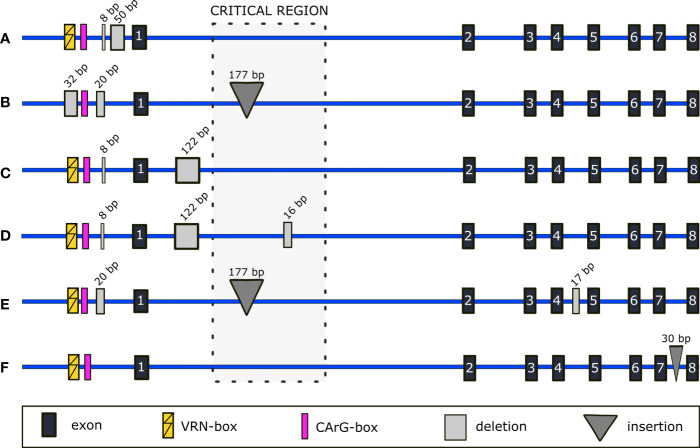
Schematic representation of selected indels detected within non-coding regions of *VRN-A1*. Promoter mutations in **(A)** correspond to known mutations of *Vrn-A1f* and *Vrn-A1f-like* alleles, but no intronic mutations are present. Mutations in **(B)** correspond to the *Vrn-A1d* allele with revealed 177-bp insertion in intron 1. Newly detected intron 1 deletion is shown in **(C)**. In **(D)**, known mutations of the *VRN-A1b* allele and novel 17-bp deletion of intron 4 are depicted. A novel deletion in intron 7 is represented in **(E)**. An insertion of 30 bp situated in intron 7 is shown in **(F)**.

**Figure 3:**
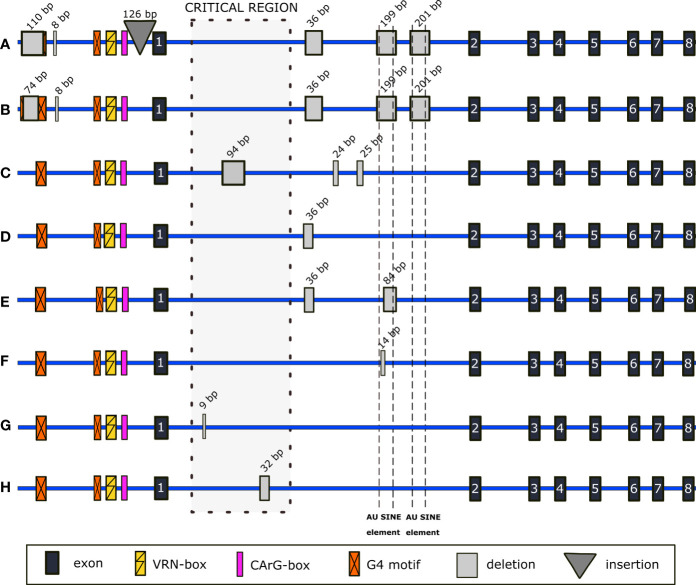
Schematic representation of selected indels detected within non-coding regions of *VRN-B1*. In **(A)** and **(C–H)**, alleles with several different new indels are shown. In **(B)**, promoter mutations correspond to mutations described for the *Vrn-G1a* allele (GenBank KX344118.1) and are combined with newly found mutations of intron 1.

**Figure 4:**
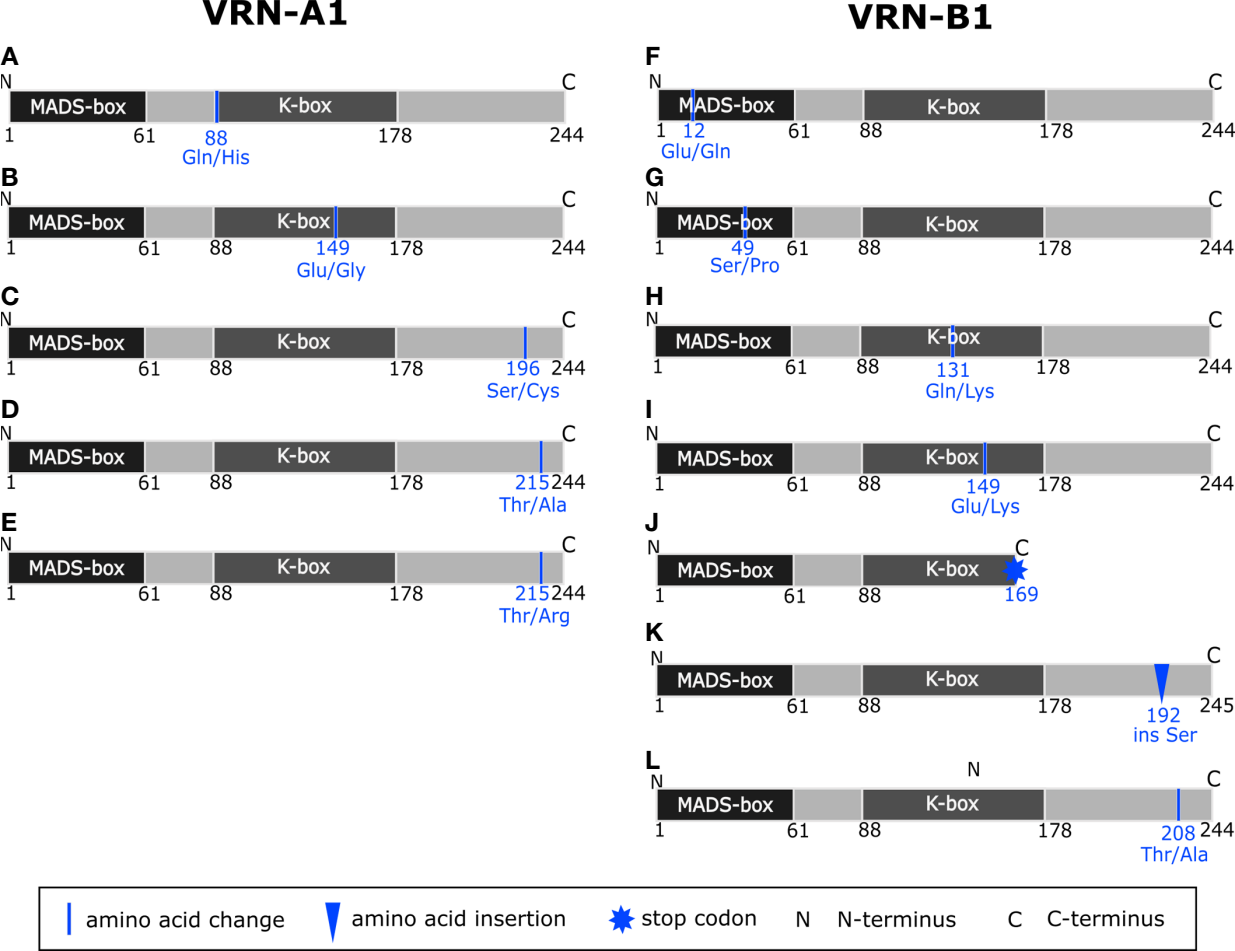
Schematic representation of predicted variants of VRN-A1 **(A–E)** and VRN-B1 **(F–L)** proteins based on the change in the nucleotide sequence of *VRN-A1* and *VRN-B1* genes, respectively. Domains were identified using the InterPro database (Mulder et al., 2007).

The full-length gene sequencing showed a significant variability within promoters, introns, and exons of both *VRN-A1* and *VRN-B1* alleles (Supplementary Material). Often, more mutations were identified within one allele, and in a number of genotypes were mutated both *VRN1* homoeoalleles. Newly found mutations of promoter and introns or their combinations are depicted in **Figures 2** and **3**. Exonic mutations are summarized in **Tables 1** and **2**, and resulting changes in VRN1 proteins are illustrated in **Figure 4**. The positions of all mutations are given from the start codon. The minus (-) sign means upstream direction.

**Table 1:** Mutations within *VRN-A1* exons. ´+´ group contains large number of genotypes (38) listed in the **Supplementary Table 13**.

Exon	Nucleotide position from ATG	Nucleotide substitution	Amino acid change	Type of change	Genotype
**Exon 1**	9	G/C	synonymous	–	PI 538709
	81	G/A	synonymous	–	PI 470956
**Exon 2**	8,757	C/T	synonymous	–	PI 466943, PI 470945, PI 466935
	8,763	A/T	synonymous	–	PI 470975, PI 470982, PI 479782, PI 466980, PI 466981
	8,781	G/C	Gln^88^/His^88^	substitution	PI 538646, PI 538648, PI 428046, PI 428051
**Exon 4**	10,410	A/G	synonymous	–	PI 538709
**Exon 5**	10,717	A/G	Glu^149^/Gly^149^	substitution	PI 470993, PI 538709
	10,720	A/G	Glu^149^/Gly^149^	substitution	PI 470956
	10,733	T/G	synonymous	–	PI 538709
**Exon 7**	11,053	C/T	Ala^180^/Val^180^	substitution	+
	11,093	A/T	synonymous	–	PI 470956, PI 470993 (TRI 18501), PI 470975, PI 470982, PI 479782, PI 656872, PI 538659, PI 560872, PI 538709, PI 466981
	11,101	C/G	Ser^196^/Cys^196^	substitution	PI 428089 (WE 27 and WE 28)
**Exon 8**	11,321	A/G	synonymous	–	PI 538709
	11,322	A/G	Thr^215^/Ala^215^	substitution	PI 538659, PI 538709
	11,323	C/G	Thr^215^/Arg^215^	substitution	PI 470975, PI 479782

**Table 2:** Mutations within *VRN-B1* exons. ´+´ group contains large number of genotypes (18) listed in the **Supplementary Table 16**.

Exon	Nucleotide position	Nucleotide substitution	Amino acid change	Type of change	Genotype
**Exon 1**	34	G/C	Glu^12^/Gln^12^	substitution	PI 487263
	105	C/T	synonymous	–	PI 428054
	145	T/C	Ser^49^/Pro^49^	substitution	PI 560872
**Exon 4**	11,391	C/A	Gln^131^/Lys^131^	substitution	PI 352322, PI 428113, PI 466990
**Exon 5**	11,640	G/A	Glu ^149^/Lys^149^	substitution	PI 428082, PI 428136, PI 428143, PI 503310
**Exon 6**	11,855	C/T	Gln^169^/*	stop codon	PI 466991
**Exon 7**	12,014	TCT ins	Ser^192^ ins	insertion	Zavitan, PI 470993, PI 428054, PI 538717
	12,062	A/G	Thr^208^/Ala^208^	substitution	+

‘*’ refers to the gain of a stop codon and ‘ins’ to an insertion of nucleotide or amino acid.

### 3.3 Sequence variability of *VRN-A1*

#### 3.3.1 Mutations of the promoter

Twenty-three wild emmer wheat genotypes (**Supplementary Table 10**) carry the *VRN-A1b* allele (Yan et al., 2004). They can be further divided according to the mutations in the VRN-box (Muterko et al., 2016) into *Vrn-A1b.2* and *vrn-A1b.4*.

The known deletion of 8 bp at the -120 bp from the start codon (Golovnina et al., 2010) was found in seven winter and three spring genotypes (**Supplementary Table 11**).

The promoter of PI 560872 carries a combination of deletions (**Figure 2A**) described in *Vrn-A1f* (GenBank GQ451751) and *Vrn-A1f-like* (GenBank KT696537) alleles (Golovnina et al., 2010; Ivaničová et al., 2016). For a more detailed comparison of the three alleles, see **Supplementary Table 12**.

The winter genotype PI 428054 carries promoter mutations (GenBank OP831152) corresponding to the *Vrn-A1d* allele (**Figure 2B**).

#### 3.3.2 Mutations within introns

As expected, we found most of the sequence variability in the intron 1. Besides SNPs, larger mutations were present in some samples. The 177-bp insertion (Strejčková et al., 2021) was detected in all genotypes with the *VRN-A1b* allele and in the genotype PI 428054 with the promoter mutation known from the *Vrn-A1d* allele. We sequenced *Vrn-A1d* from Israeli *T. dicoccoides* line Tabigha 15 (provided by The Institute of Evolution Wild Cereal Gene Bank at the University of Haifa), which was characterized only at the promoter sequence to date (Yan et al., 2004). Indeed, the *Vrn-A1d* allele from Tabigha 15 also contains the 177-bp insertion in the intron 1 (GenBank OP831151), as detected in PI 428054.

A newly found 122-bp deletion in the intron 1 (**Figures 2C, D**) was present only in genotypes carrying allele with an 8-bp deletion in the promoter and A11093T SNP in exon 7 (Ser^196^/Cys^196^). A 16-bp deletion was found in winter genotypes PI 470956 and PI 470993 (TRI 18501) (**Figure 2D**). Most of the genotypes possess the RIP3 1_SNP haplotype GGACC (Kippes et al., 2018), besides PI 466989 carrying RIP3 3_SNP haplotype CGACT and PI 538709 with the canonical RIP3 motif GGATC (Kippes et al., 2018).

A 17-bp deletion in the intron 4 of the *VRN-A1b* allele was found in four spring genotypes (PI 538698, PI 428015 (TRI 18481), PI 538697, and PI 538696) (**Figure 2E**).

We found a 30-bp insertion in the intron 7 of three winter genotypes (PI 466958, PI 538657, and PI 428055). This insertion was a duplication of an adjacent downstream sequence (**Figure 2F**).

#### 3.3.3 Mutations involving coding sequence

Surprisingly, eight synonymous and seven non-synonymous SNPs were found within the exons 1, 2, 4, 5, 7, and 8 (**Table 1** and **Figure 4**). Only non-synonymous SNPs will be described further in the text. Synonymous SNPs are summarized in **Table 1**.

In exon 2, we detected one non-synonymous SNP G8781C changing the amino acid (AA) from glutamine into histidine (Gln^88^/His^88^) in the K-box of VRN-A1 protein (**Figure 4A**). This SNP was detected exclusively in four winter late-flowering genotypes.

In exon 5, A10717G and A10720G SNPs causing the same amino acid substitution from glutamic acid to glycine (Glu^149^/Gly^149^) in the K-box (**Figure 4B**) were found. The first SNPs were detected in two and one winter genotype, respectively.

C11053T SNP in exon 7 (Ala^180^/Val^180^) (Díaz et al., 2012) was present in 38 winter genotypes (**Supplementary Table 13**). This allele is referred to as *vrn-A1b* and is associated with higher vernalization requirement and later flowering (Li et al., 2013). The so far unpublished non-synonymous SNP C11101G resulting in a change of serine into cysteine (Ser^196^/Cys^196^) in the C-terminal of the VRN-A1 protein (**Figure 4C**) was revealed in two winter genotypes in combination with Ala^180^/Val^180^.

Two non-synonymous SNPs; A11322G (Thr^215^/Ala^215^) and C11323 (Thr^215^/Arg^215^) affecting same amino acid of the C-terminus (**Figures 4D, E**), were detected in exon 8. Mutations of exon 8 were rare in our analyzed set of wild emmer wheat, emerging in four winter genotypes only.

All amino acid substitutions also change the predicted secondary structure of the VRN-A1 protein (**Supplementary Figure 3A**).

### 3.4 Sequence variability of *VRN-B1*

#### 3.4.1 Mutations of the promoter

In 35 genotypes, we found SNP in the 23-bp G4 motif situated at -274 bp (**Supplementary Table 14**). PI 560872 and PI 656872 genotypes carried a 110-bp (spanning across the G4 motif, position -735 bp) and 8-bp deletion (-583 bp), together with 126-bp insertion (-100 bp) (**Figure 3A**) (GenBank OP831155). Golovnina et al. (2010) reported an identical transposon insertion at -100 bp (GenBank GQ451771.1). In PI 538659, promoter deletions identical to that described for *Vrn-G1a* (GenBank KX344118.1) (Shcherban et al., 2016) were detected (**Figure 3B**). Interestingly, the G4 motif is reconstituted by the adjacent two bp (**Supplementary Figure 4**). Contrary to its *VRN-A1* homoeoallele, there were no mutations within the VRN- and CArG-box.

#### 3.4.2 Mutations within introns

*VRN-B1* intron 1 shows large sequence variability, including deletions of various lengths. In spring genotypes PI 538691 and PI 538695, the combination of 94-bp, 24-bp, and 25-bp deletions was detected (**Figure 3C**) (GenBank OP831153 and OP831154). We detected different deletions covering the Au SINE elements of *VRN-B1* intron 1: complete 199- and partial 84- and 14-bp deletions of the first Au SINE and complete 201-bp deletion of the second one. Opposite to the complete deletions, partial deletions were found across a wide range of winter genotypes, from early to late flowering ones, as well as in spring. Several genotypes share a 36-bp deletion (**Figure 3D**) (PI 467001), mainly together with an 84-bp deletion (**Figure 3E**) (PI 428082, PI 352322, PI 428113, PI 428136 (TRI 18489), PI 428143, CItr 17675, PI 503310 and PI 466990), or 199- and 201-bp deletions (**Figures 3A** **, E**) (PI 656872, PI 538659 and PI 560872) spanning over the Au SINE elements. Another common mutation of intron 1 found in six out of 95 sequenced genotypes was the deletion of 14 bp located in the Au SINE element (**Figure 3F** and **Supplementary Table 15**). A 9-bp deletion was found only in the winter genotype PI 466961 (**Figure 3G**) and a 32-bp deletion in the winter genotype PI 428043 (**Figure 3H**).

#### 3.4.3 Mutations involving coding sequence

In exons 1, 4, 5, 6, and 7 (**Table 2** and **Figure 4**) of *VRN-B1*, seven different non-synonymous and one synonymous SNP were found. Only non-synonymous SNPs will be described in the text below. Synonymous SNPs are summarized in **Table 2**.

In exon 1 were detected the G34C SNP (MADS-box, Glu^12^/Gln^12^) (**Figure 4F**) and the T145C SNP, resulting in the Ser^49^/Pro^49^ change in the MADS-box of VRN-B1 protein (**Figure 4G**). Each of the mentioned SNPs was unique.

In exon 4 of two winter and one spring genotypes, C11391A SNP was found, causing AA substitution Gln^131^/Lys^131^ in the K-box of VRN-B1 (**Figure 4H**).

Another SNP, G11640A, causing AA substitution within the K-box (Glu^149^/Lys^149^), was detected in exon 5 of four winter genotypes (**Figure 4I**).

An interesting mutation resulting in premature stop codon was found in exon 6 (C11855T) of PI 466991. The predicted VRN-B1 protein is only 169 AA long and contains an intact MADS-box and partial K-box (**Figure 4J**).

Insertion of TCT into exon 7, causing insertion of Ser^192^ in the VRN-B1 protein, is present in four winter genotypes (**Figure 4K**). AA change Thr^208^/Ala^208^ encoded by a mutation in exon 7 (A12062G) was found exclusively in 13 early flowering winter genotypes and five spring genotypes (**Supplementary Table 16** and **Figure 4L**).

All amino acid substitutions, except Lys^131^/Gln^131^ in the K-box, change the predicted secondary structure of the VRN-B1 protein (**Supplementary Figure 3B**).

### 3.5 Gene expression of selected *VRN-A1* and *VRN-B1* alleles

**Figure 5:**
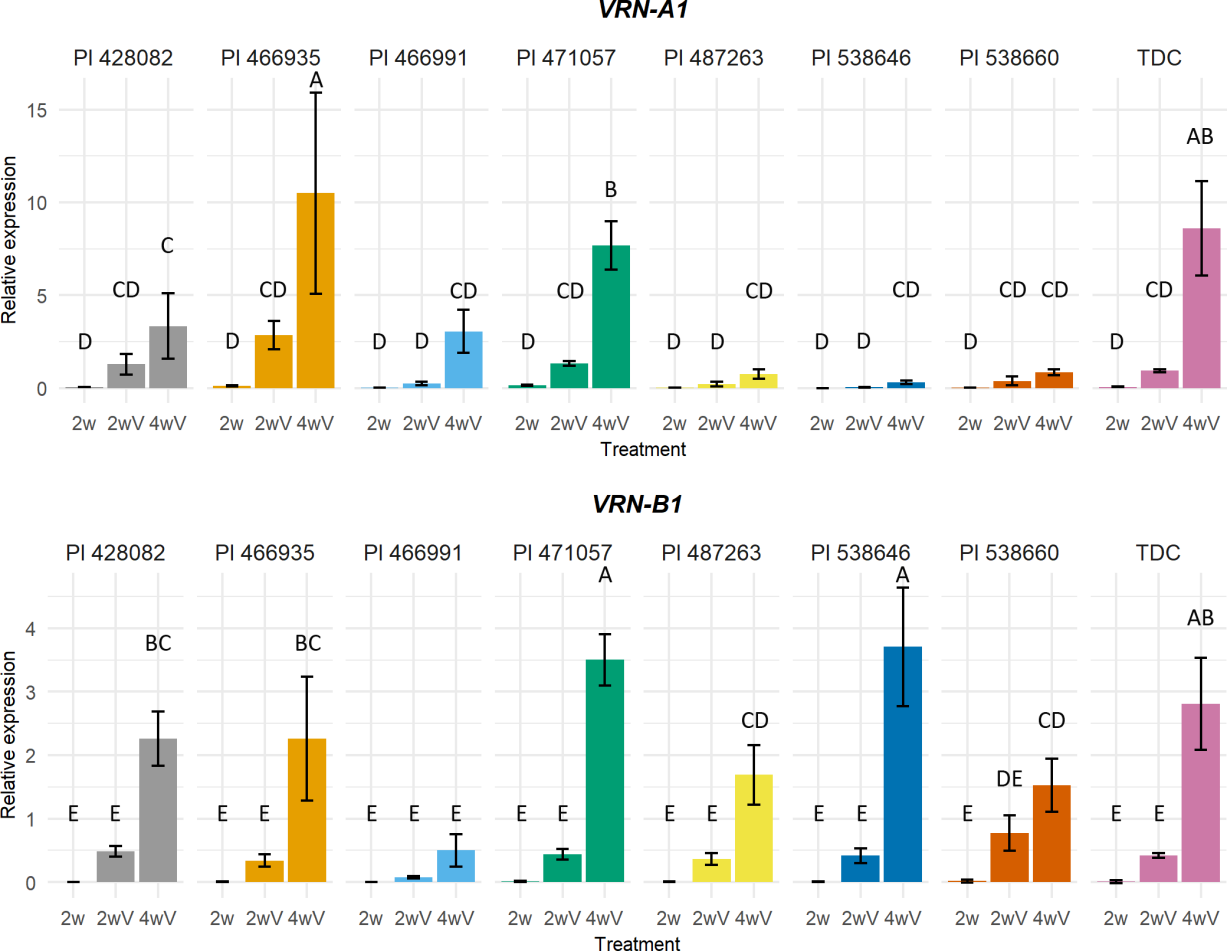
*VRN-A1* and *VRN-B1* expression profiles of PI 428082, PI 466935, PI 466991, PI 471057, PI 487263, PI 538646, PI 538660, and TDC. Expression data were analysed using one-way ANOVA with Duncan’s post-hoc test at α=0.05. Means that do not share a letter are statistically different. 2w – two weeks under 21°C LD, 2wV - two weeks under 21°C LD, followed by two weeks of vernalization, 4wV - two weeks under 21°C LD, followed by four weeks of vernalization.

Six different genotypes (PI 428082, PI 466935, PI 466991, PI 471057, PI 487263, PI 538646, PI 538660) were chosen for the RT-qPCR experiment to assess the influence of some of the detected VRN1 mutations. We compared those genotypes with the bread wheat cultivar TDC and the late flowering PI 538660. TDC has the intact recessive *VRN1* alleles, while PI 538660 possesses the *vrn-A1b* allele (C11053T SNP, Ala^180^/Val^180^) and intact *vrn-B1* (**Figure 5**). The *vrn-A1b* allele showed to be the most abundant recessive allele in the sequenced panel.

After four weeks of vernalization treatment, the highest *VRN-A1* expression was detected in the early flowering winter genotype PI 466935 with synonymous C8757T mutation in exon 2. On the contrary, the expression of late flowering PI 538646 with Gln^88^/His^88^ substitution in VRN-A1 K-box was very low. It was similar to PI 538660 and PI 487263, carrying the *vrn-A1b* allele for a stronger vernalization requirement. The expression level of PI 471057 intact *vrn-A1* was similar to TDC, while in PI 428082 intact *vrn-A1* showed lower expression compared to TDC.

The *vrn-B1* allele from PI 466935 and the *vrn-B1* allele from PI 428082 have the same expression profile. The *vrn-B1* allele in PI 466935 has a small 6-bp deletion in intron 7. The *vrn-B1* allele in PI 428082 carries intron 1 deletions and substitution in the K-box. The expression of PI 466991 *vrn-B1* containing nonsense mutation was minimal. PI 471057 *vrn-B1* with C-terminal substitution and PI 538646 *vrn-B1* with 7-bp intron 1 insertion (expansion of microsatellite repeat ACCCCCC) expression was the highest. PI 487263 *vrn-B1* with MADS-box substitution reached the same level as intact PI 538660 *vrn-B1.*

### 3.6 Distribution of *VRN1* variants across the Fertile Crescent

**Figure 6:**
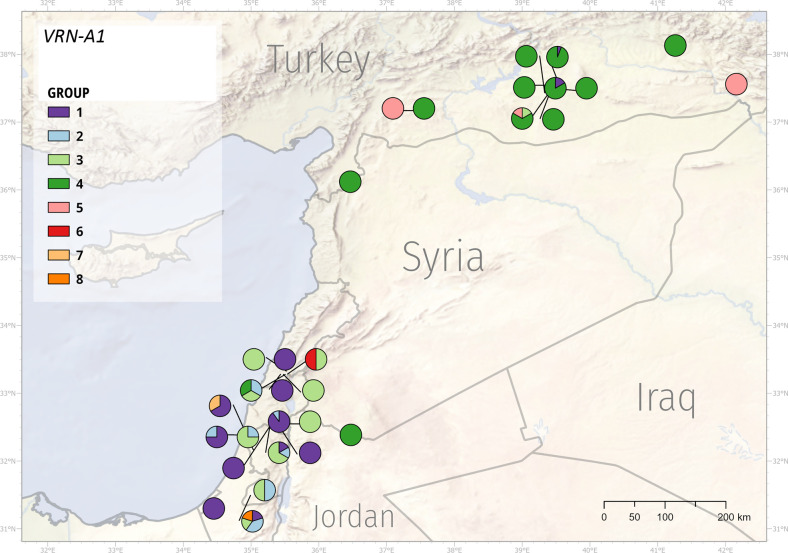
Geographic distribution of the *VRN-A1* alleles. WEW genotypes formed eight groups according to the *VRN-A1* mutations.

**Figure 7:**
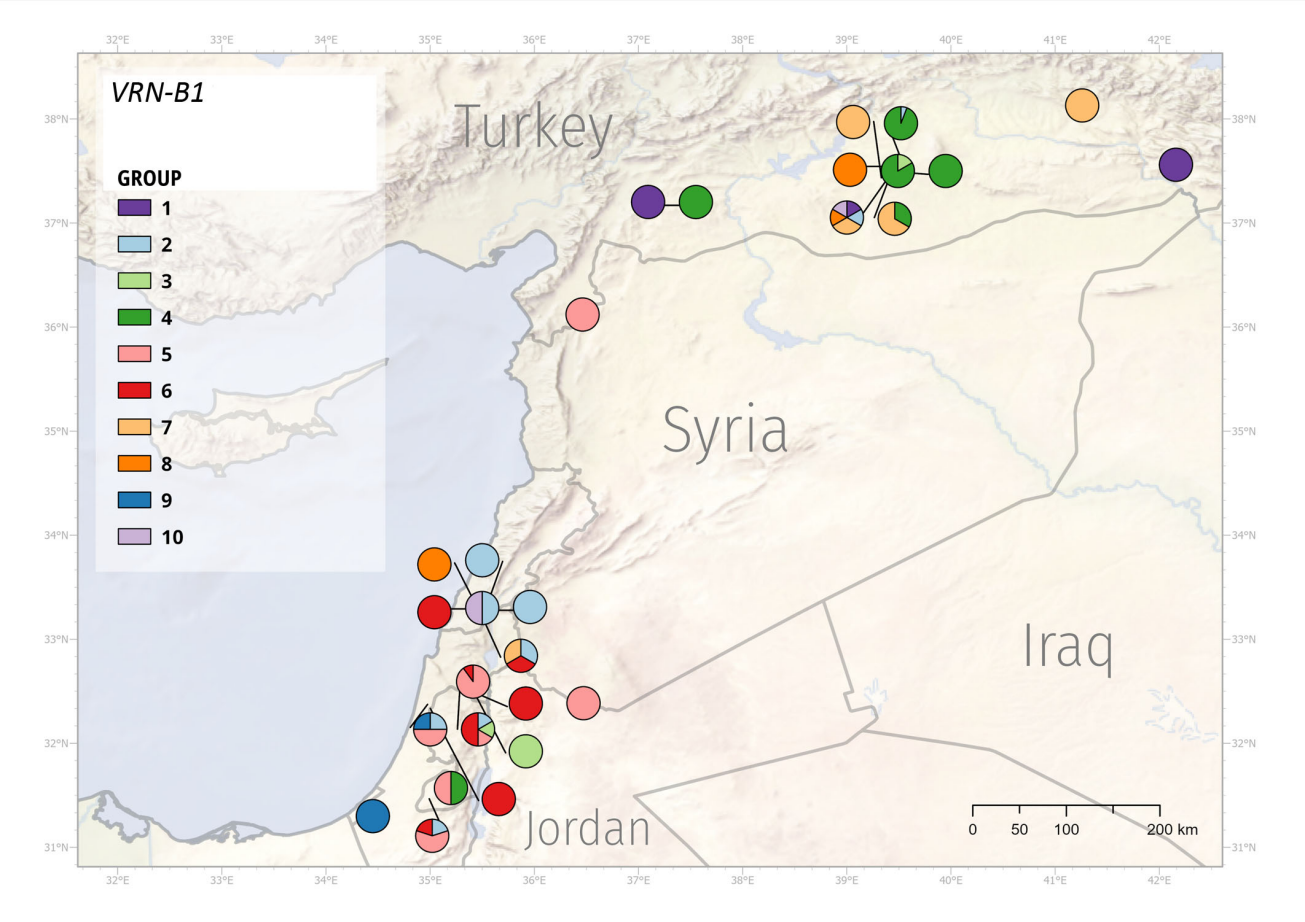
Geographic distribution of the *VRN-B1* alleles. WEW genotypes formed 10 groups according to the *VRN-B1* mutations.

Subsequently, we inspected the distribution of identified *VRN1* variants across the Fertile Crescent. Because of the high sequence diversity across the sequenced panel, we decided to construct a phylogenetic tree for both *VRN-A1* and *VRN-B1* sequences (**Supplementary Figures 5** and **6**). We used resultant individual clades (further referred to as ‘group’) and mapped individuals from each group to their collection site to illustrate the distribution of *VRN1* alleles (**Figures 6** and **7**).

Based on the *VRN-A1* sequences, genotypes were split into eight groups (**Supplementary Table 17**). We could not obtain the full-length *VRN-A1* sequence of PI 466988 and PI 538691. Therefore, we did not include these samples in the phylogenetic analysis. However, based on their partial sequences and genotyping, they most likely belong to groups 1 and 2, respectively. Group 1 contains all 23 genotypes with the *VRN-A1b* allele and one genotype with the *Vrn-A1d* allele. Group 2 includes nine genotypes with 122-bp deletions in intron 1 with combinations of two different mutations in exon 2, and 7. Group 3 contains alleles with high sequence similarity to the intact *vrn-A1* allele. Group 4 is the most numerous one and covers all genotypes (38) with the exon7 C11053T SNP. This SNP results in amino acid change Ala^180^/Val^180^ associated with stronger vernalization requirement (Díaz et al., 2012; Li et al., 2013). Out of the 38 genotypes, 35 come from Turkey. Group 5 is formed by three genotypes from Turkey containing the A11093T SNP in exon 7. Groups 6-8 contain only one genotype each and cover genotypes with a very high number of SNPs, *Vrn-A1c* allele, and RIP3_3SNPs haplotype, respectively.

*VRN-B1* sequence similarity divided genotypes into ten groups (**Supplementary Table 18**). Genotypes in group 1 share 36-, 199- and 201-bp deletions of intron 1. Group 2 genotypes possess 36- and 84-bp deletion in intron 1, besides PI 467001 carrying only 36-bp deletion. Genotypes in group 3 share 3-bp insertion in exon 7. Group 4 harbors 25 genotypes with intact *vrn-B1* and PI 428043 with a 32-bp deletion in intron 1. Group 5 contains all 18 genotypes with A12062G SNP in exon 7, PI 487260, and PI 487263. Group 6 is very diverse, with 15 genotypes. Group 7 includes eight genotypes with intact *vrn-B1*. Group 8 contains only three genotypes, and groups 9 and 10 contain two genotypes each.

## 4 Discussion

Tetraploid durum wheat (*T. turgidum* L. ssp. *durum* (Desf.) Husn.) and hexaploid bread wheat (*T. aestivum* L.) are important crops grown on 220 million hectares, which is more than any other food crop (Foreign Agricultural Service/USDA, 2022). Both wheats share a common progenitor, wild emmer wheat (*T. turgidum* ssp. *dicoccoides* (Schrank ex Schübl.). Following the domestication in the Fertile Crescent, wheat evolved along ancient human migration roads and adapted to a wide range of agro-climatic regions (Balfourier et al., 2019). This was made possible by controlling the flowering time depending on the growing conditions. The flowering time is influenced by photoperiod and temperature. Wheat genotypes can be classified as winter, facultative or spring based on the requirement of a cold period to switch from the vegetative to the reproductive phase (vernalization). We studied a collection of 263 winter and spring WEWs from Turkey, Lebanon, Syria, Palestina, Israel, Iraq, and Iran, covering the Fertile Crescent region. In this collection, we observed heading time differences and identified alleles lost in modern wheat during the breeding process. Such alleles might be beneficial in breeding programs.

### 4.1 HD loci in a winter wild emmer population

We performed GWAS on 178 WEW genotypes and found 16 MTAs (Marker-trait associations) associated with heading time (**Supplementary Table 7**) The candidate genes were annotated based on the best Uniprot balstp hit (**Supplementary Material 1**).

Three candidate genes for AX-94761084 on chromosome 1B (*TRIDC1BG067780*, *TRIDC1BG067750*, and *TRIDC1BG067760*) encode product with sequence homology to *Arabidopsis* BTB/POZ and MATH domain-containing protein 3 (BPM3). BPMs act as substrate-specific adapters for E3 ubiquitin-protein ligase complex (Chen et al., 2013), interacting with the negative regulator of flowering, MYB56, at the promoter of the floral activator, *FLOWERING LOCUS T* (Chen et al., 2015).

The most interesting candidate gene for QTL associated with AX-95219400 SNP on 3A is *TRIDC3AG046200*, with similarity to *Arabidopsis ULTRAPETALA1* (*ULT1*), a trithorax group factor regulating transcriptional activation and counteracting EMF1 repressor function (Carles et al., 2005; Carles and Fletcher, 2009; Pu et al., 2013).

One of the candidate genes for AX-94918249 on chromosome 5A is *TRIDC5AG000640*, similar to *Oryza sativa EARLY FLOWERING1* (*EL1*). It codes for CASEIN KINASE 1-LIKE protein HD16 involved in the regulation of flowering time through gibberellin signaling and photoperiod pathway under LD conditions (Hori et al., 2013; Kwon et al., 2015). AX-94918249 belongs to the QTL region QHd.cerz-5AS.1 associated with heading time, previously reported in *T. turgidum* L. ssp. *durum* (Roncallo et al., 2017).

An interesting candidate gene for another heading time-associated marker (AX-94609685) located on chromosome 5A is *TRIDC5AG070010*, similar to *NAC domain-containing protein 37.* NAC-domain protein LONG VEGETATIVE PHASE 1 (LOV1) was found to integrate and regulate flowering time and cold response in *Arabidopsis* through repression of *CO* under LD (Yoo et al., 2007). AX-94609685 is included in the QTL region QHd.cerz-5AL.3 associated with heading time in *T. turgidum* L. ssp. *durum* (Roncallo et al., 2017).

A candidate gene *TRIDC6BG043170* on chromosome 6B (AX-94853271) encodes a protein similar to Cold-responsive protein kinase 1 that phosphorylates proteins and triggers their translocations from the cytosol to the nucleus in response to cold stress (Liu et al., 2017). Studies in wheat reported that cold-induced phosphorylation of VER2 is necessary for the VER2 transport from cytoplasm to nuclei, where it interacts with vernalization repressor GRP2 and releases the *VRN1* pre-mRNA (Xing et al., 2009; Xiao et al., 2014; Kippes et al., 2018).

A possible candidate gene for AX-95172810 on chromosome 7B might be *TRIDC7BG000850*, similar to *Arabidopsis* B-box zinc finger protein 22. An example of a well-known *Arabidopsis* B-box zinc finger protein is CONSTANS (CO), promoting flowering under LD conditions (Putterill et al., 1995). One of the candidate genes is also *Glutathione S-transferase T1* (*GSTT1*, *TRIDC7BG000060*), which homologs were identified as candidates for QTL related on heading date in the 7B chromosome arm substitution line of WE chromosome 7B in the genetic background of hexaploid Chinese Spring (Lu et al., 2022). It is well known that the *GST* is linked with diverse abiotic stresses. For instance, eighty-four *GST* genes were identified in barley and associated with an important role in drought tolerance (Rezaei et al., 2013). Even more (330) *GST* genes were identified in hexaploid wheat, and expression profiling of 14 selected genes indicated *GST* could respond to abiotic stress (Wang et al., 2019). In addition, four genes were upregulated under cold (Wang et al., 2019). Later, the number of *GST* genes identified in *T. aestivum* increased to 346 (Hao et al., 2021). *GSTs* were also found to be upregulated in response to cold in pepper (*Capsicum annuum* L.) (Islam et al., 2019) and pumpkins (*Cucurbita maxima*) (Abdul Kayum et al., 2018).

AX-95632383 located on chromosome 7B is included in the heading time-associated QTL QHd.cerz-7BL.3 from *T. turgidum* L. var. *durum* (Roncallo et al., 2017). We detected 15 candidate genes, but none have been reported to play a role in flowering.

### 4.2 *VRN1* sequence variability

Of the four known vernalization response genes, the *VRN1* gene is considered the most important one (Yan et al., 2003). Sequence analysis of the *VRN1* gene in bread wheat revealed only minor sequence variation (Strejčková et al., 2021; Makhoul et al., 2022). This may reflect the limited gene pool of progenitors, which contributed to the original population(s) of bread wheat. To discover new alleles for potential use in the breeding of durum and bread wheat adapted to climate change, we sequenced the full-length *VRN1* genes of wild emmer wheat genotypes collected in different areas of Fertile Crescent. Former studies investigating the *VRN1* variability in wild emmer wheat relied on genotyping, restriction assays, and sequencing of short PCR products of the promoter or the first intron of *VRN1* (Yan et al., 2004; Golovnina et al., 2010; Chhuneja et al., 2015; Shcherban et al., 2015; Konopatskaia et al., 2016).

Overall, high sequence variability was found in the panel of 95 wild emmer wheat genotypes. Interestingly, mutations were found in both non-coding and coding parts of the *VRN1* gene. In total, we found 15 different SNPs in exons of *VRN-A1* and seven SNPs, and one insertion in exons of *VRN-B1*. In *VRN-A1*, seven out of the 15 SNPs were non-synonymous, resulting in the amino acid sequence change. On the contrary, seven of the eight *VRN-B1* exonic mutations changed the amino acid sequence. Gln^88^/His^88^ change in the K-box of VRN-A1 protein seems to be associated with later flowering. It was found in four Turkish winter genotypes (147-151 and 159-163 days to heading when scored in the field experiment in Turkey and Italy, respectively) with no other SNPs detected either in *VRN-A1* or *VRN-B1* alleles. Synonymous *VRN-A1* exon2 SNP C8757T was identified only in three early winter genotypes carrying the *VRN-A1b* allele (134-139 (TUR) and 150-154 (IT) days to heading). Thr^208^/Ala^208^ amino acid change of VRN-B1 was found only in 13 early winter and five spring genotypes.

*VRN1* alleles respond differently to environmental stimuli and have adaptive value to specific environments (Stelmakh, 1998). The observed sequence mutations within the *VRN-A1* and *VRN-B1* alleles formed eight and ten groups, respectively. Group 4 (*VRN-A1* allele) was predominantly represented by WEW from Turkey, although several Turkey’s genotypes were found in other groups as well. Similarly, group 1 (*VRN-A1* allele) mainly included the genotypes from Syria and Israel (**Figure 6**, **Supplementary Table 19**). The geographic distribution of *VRN-B1* groups also showed that the vast majority of Turkey’s genotypes fell into the same group (**Figure 7**, **Supplementary Table 20**). These results suggest that genotypes clustering in the same group may share a pedigree and possibly a common ancestor. Although the frequencies of genotypes with different *VRN1* alleles in different geographical regions suggest a possible adaptive role, additional studies using larger sample size will be necessary to quantify better the effect of the *VRN1* alleles on the adaptation to different environments.

#### 4.2.1 Partially characterized VRN1 alleles

Due to the limited sequencing technologies in the past, several *VRN1* alleles are characterized only by a short partial promoter or intronic sequence. As shown before (Strejčková et al., 2021), the known allele *VRN-A1b* characterized by the 20-bp deletion in the promoter region (Yan et al., 2004) also carries a 177-bp insertion in the first intron. In our study, we found the same 177-bp insertion in the *Vrn-A1d* allele of PI 428054 (GenBank OP831152) and Tabigha 15 (GenBank OP831151), characterized so far only by 32-bp and 20-bp deletions in the promoter (Yan et al., 2004). Konopatskaia et al. (2016) proposed two possible mechanisms of *Vrn-A1d* origin: (1) from an extension of deletion in the *VRN-A1b* allele or (2) through the formation of two deletions in the *vrn-A1* allele. Our data support the first hypothesis because both *Vrn-A1d* and *VRN-A1b* alleles share the 177-bp insertion in the intron 1.

The PI 560872 *VRN-A1* allele shows high similarity with spring *Vrn-A1f* and *Vrn-A1f-like* alleles (Golovnina et al., 2010; Ivaničová et al., 2016) but lacks any indels in the intron 1, as described for *Vrn-A1f-like.* Unfortunately, the full-length sequence of *Vrn-A1f* is not available. Despite described mutations in the *VRN-A1* promoter, the growth type for PI 560872 is determined as winter according to the GRIN-Global database (Version: 2.0.5.0), further supported by the heading time experiments. One can hypothesize that not the promoter variation but the intron 1 mutations are responsible for the spring phenotype in the *Vrn-A1f* and *Vrn-A1f-like* alleles. Civáň et al. (2013) and Badaeva et al. (2022) classified PI 560872 as *T. timopheevii* ssp. *armeniacum*.

#### 4.2.2 Premature stop codon

The keratin-like (K-box) domain for VRN1 protein dimerization is postulated to form three amphipathic α-helices K1, K2, and K3 (Ma et al., 1991; Yang et al., 2003). Our study found a VRN-B1 protein with premature stop codon causing partial loss of K3 in the K-domain and complete loss of the C-terminal domain. Despite the mutation, PI 466991 was able to flower on average in 142 and 160 days when scored in the field experiments in Turkey and Italy, respectively. When grown under controlled conditions without vernalization treatment, only one out of seven plants survived and flowered in 199 days (data not shown). We can speculate that a functional *VRN-A1* copy is sufficient for the transition into the reproductive stage. Moreover, the Δ*vrn1*-null mutants demonstrated that complete *VRN1* is not essential for flowering (Chen and Dubcovsky, 2012). Premature termination codon (PTC) can affect protein biosynthesis, gene expression, or mRNA stability (Gao et al., 2021; Liu et al., 2022). Transcripts of the mutant gene can be processed by nonsense-mediated mRNA decay, a mechanism for detecting and rapidly eliminating PTC-containing mRNAs (Maquat, 1995; Frischmeyer and Dietz, 1999; Hentze and Kulozik, 1999). Interestingly, the *VRN-B1* expression level of other winter genotypes was significantly higher after four weeks of vernalization than the *VRN-B1* expression level in PI 466991 (**Figure 5**). However, it is impossible to separate the effect of the PTC on the *VRN-B1* expression from the mRNA stability.

#### 4.2.3 Expression of mutant VRN1 alleles

The promoter region of the *VRN1* gene contains multiple regulatory sites, and it is sufficient to induce transcription (Yan et al., 2004; Alonso-Peral et al., 2011). However, additional regulatory sites were found outside the promoter region (Fu et al., 2005). Our study identified several VRN1 amino acid substitutions in early and late flowering genotypes.

The gene expression analysis showed various effects of discovered mutations on transcript levels. Some mutations did not change the *VRN-A1* expression, for instance, VRN-A1 K-box Gln^88^/His^88^ substitution in PI 538646 *vrn-A1*. Its expression was comparable to the *vrn-A1b* allele in the late flowering genotypes.

The PI 466935 *VRN-A1b* allele containing synonymous C8757T substitution in exon 2 showed the highest expression level. This mutation was found only in early flowering winter genotypes. The expression level of the *vrn-B1* allele containing a 6-bp deletion in intron 7 was comparable to PI 466935.

Thr^208^/Ala^208^ substitution in the C-terminus of VRN-B1 of PI 471057 is associated with earlier winter and spring genotypes. Its expression was very high, increasing rapidly from two to four weeks of vernalization. It seems it also affects the expression of intact *vrn-A1.*

According to the currently accepted version of the ‘original’ vernalization model, activated *VRN1* represses the flowering repressor *VRN2* and upregulates the flowering activator *FT*, which further promotes the *VRN1* expression (Chen and Dubcovsky, 2012; Debernardi et al., 2022). Tanaka et al. (2018) hypothesize that VRN1 binds the *FT* promoter directly and suggest that the late flowering phenotype of wheat with Ala^180^/Val^180^ substitution in VRN-A1 (Díaz et al., 2012; Li et al., 2013) might be caused by lower binding affinity of the changed protein to the *FT* promoter. This finding could explain the above-discussed changed expression of *VRN1* alleles with exonic mutations or even altered expression of the intact homoeoallele. Based on the analysis of the VRN1 K-box, it is hypothesized that VRN1 acts as a tetramer (Itoh et al., 2021). Amino acid changes in the K-box and C-terminus responsible for the higher-order formations might hypothetically affect the tetramer formation and the binding affinity of the final VRN1 complex.

#### 4.2.4 G4 motif deletions and Au SINE elements

G-quadruplex (G4) secondary structures can participate in the regulation of many cellular processes. Several studies in animals observed the direct role of G4 motifs in the regulation of replication, transcription, and mi-RNA expression (Huppert and Balasubramanian, 2007; Maizels and Gray, 2013; Valton et al., 2014; Lago et al., 2021; Spiegel et al., 2021; Zhu et al., 2021). In promoters of plants, strong co-occurrence of G4 motifs with transcription factors binding sites such as Telobox, MYB, and E2F motif was detected (Garg et al., 2016). Evidence of G4 motif significance in regulating gene expression in plants is emerging (Yang et al., 2020; Volná et al., 2021; Feng et al., 2022). Almost one million different G4 motifs were identified in bread wheat, enriched at the transcription start sites (TSS), first coding domain sequence (CDS), and the start codon (Cagirici and Sen, 2020). Cagirici and Sen (2020) also suggested a potential role of G4 in regulating *VRN1* genes. The *VRN-B1* promoter of two of the latest flowering winter genotypes (PI 560872 and PI 656872) carries the deletion of G4 in the *VRN-B1* promoter. PI 560872 exhibits extremely late flowering when grown without vernalization in controlled conditions (43 days later than non-vernalized TDC, data not shown). Interestingly, it also possesses deletions in the *VRN-A1* promoter known from spring alleles *Vrn-A1f* and *Vrn-A1f-like*, which have no early flowering effect in the PI 560872 genotype. Besides the deletion of the G4 motif in PI 560872 and PI 656872, there are also two complete Au SINE elements deletions in the intron 1 of *VRN-B1*, which could have a potentially negative effect on the flowering time since they can also be found in another late flowering genotype, PI 538659. Au SINE elements are non-long terminal repeat retrotransposons, which may affect the gene structure and function as they are able to create allelic variation (Keidar et al., 2018).

In conclusion, due to climate change and the growing human population, there will be a need to breed new cultivars that are more resilient to abiotic stress with higher yields. The wild emmer wheat genotypes provide additional variability for the flowering time response (Leigh et al., 2022). Here, we deliver diverse full-length sequences of the critical player of vernalization, the *VRN1* gene. Our comprehensive sequence and expression analysis of the *VRN1* alleles in the set of wild genotypes from Fertile Crescent provides an available reservoir of allelic diversity that could be introduced into breeding programs to expand the elite wheat gene pool. Moreover, the genome-wide association study (GWAS) results indicate other potential candidates for the fine-tuning of the flowering time.

## Data availability statement

The datasets presented in this study can be found in online repositories. The names of the repository/repositories and accession number(s) can be found in the article/**Supplementary Material**.

## Author contributions

JŠ and HÖ designed the study. BS conducted laboratory experiments. RČ performed GWAS analysis. BS, ZM, and RČ analyzed the data. ZM carried out a statistical analysis. BS, JŠ, ZM, and RČ wrote the manuscript. EM and HÖ developed, genotyped and characterised the wild emmer wheat collection, EÇ and AMM, phenotyped the collection in field experiments. JB analyzed the geographical distribution of *VRN1* alleles. JŠ supervised the results. All authors contributed to the article and approved the submitted version.
