# Supplementary material for: Wild emmer wheat, the progenitor of modern bread wheat, exhibits great diversity in the VERNALIZATION1 gene
Source: Front Plant Sci. 2023 Jan 6;13:1106164. doi: 10.3389/fpls.2022.1106164 (PMC9853909; doi:10.3389/fpls.2022.1106164)
Supplement: Supplementary file 1 [file DataSheet_1.pdf]

Supplementary Material

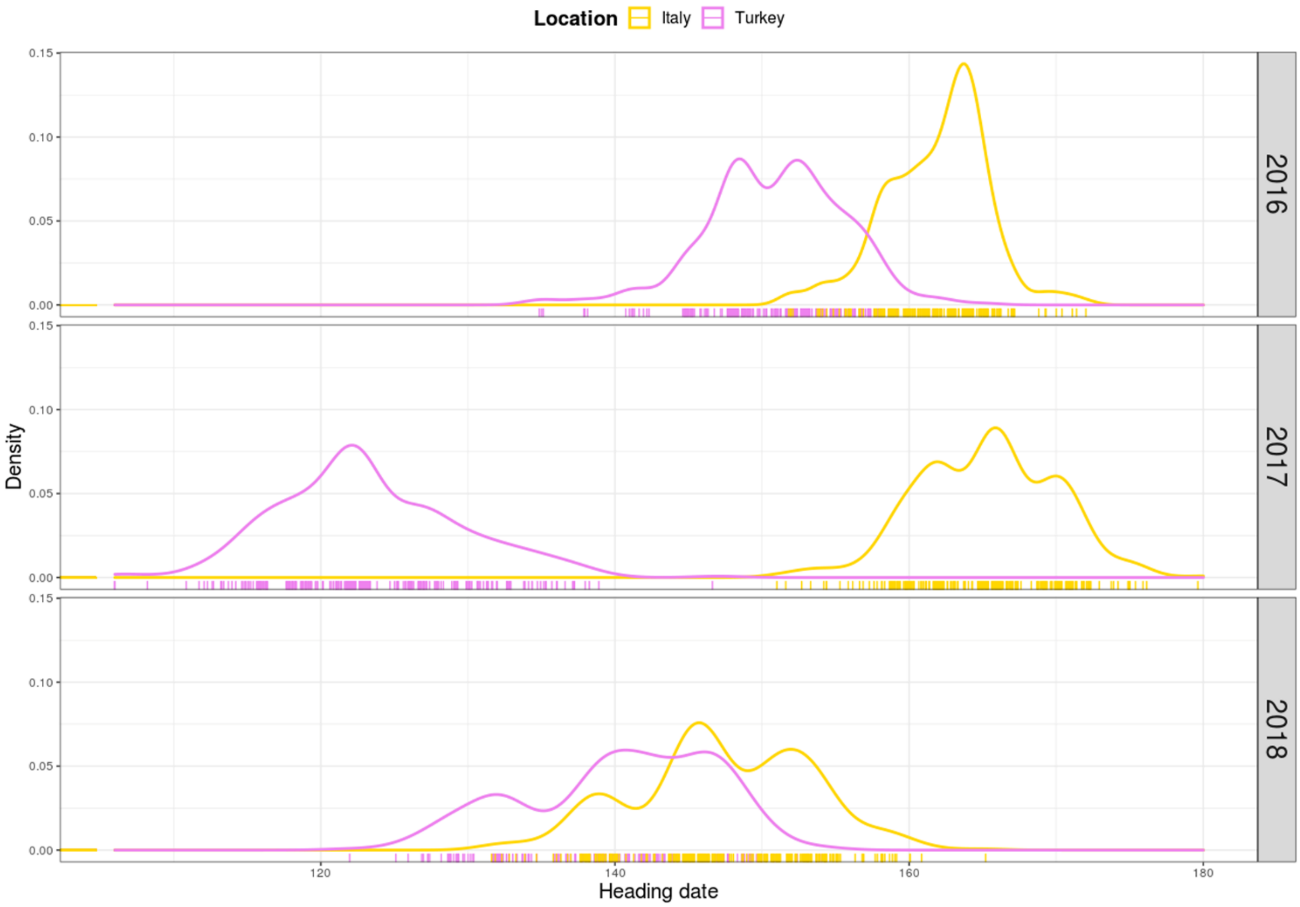

Supplementary Figure 1. Density histograms of observed phenotypic data from Italy and Turkey during three seasons (2016-2017, 2017-2018 and 2018-2019).

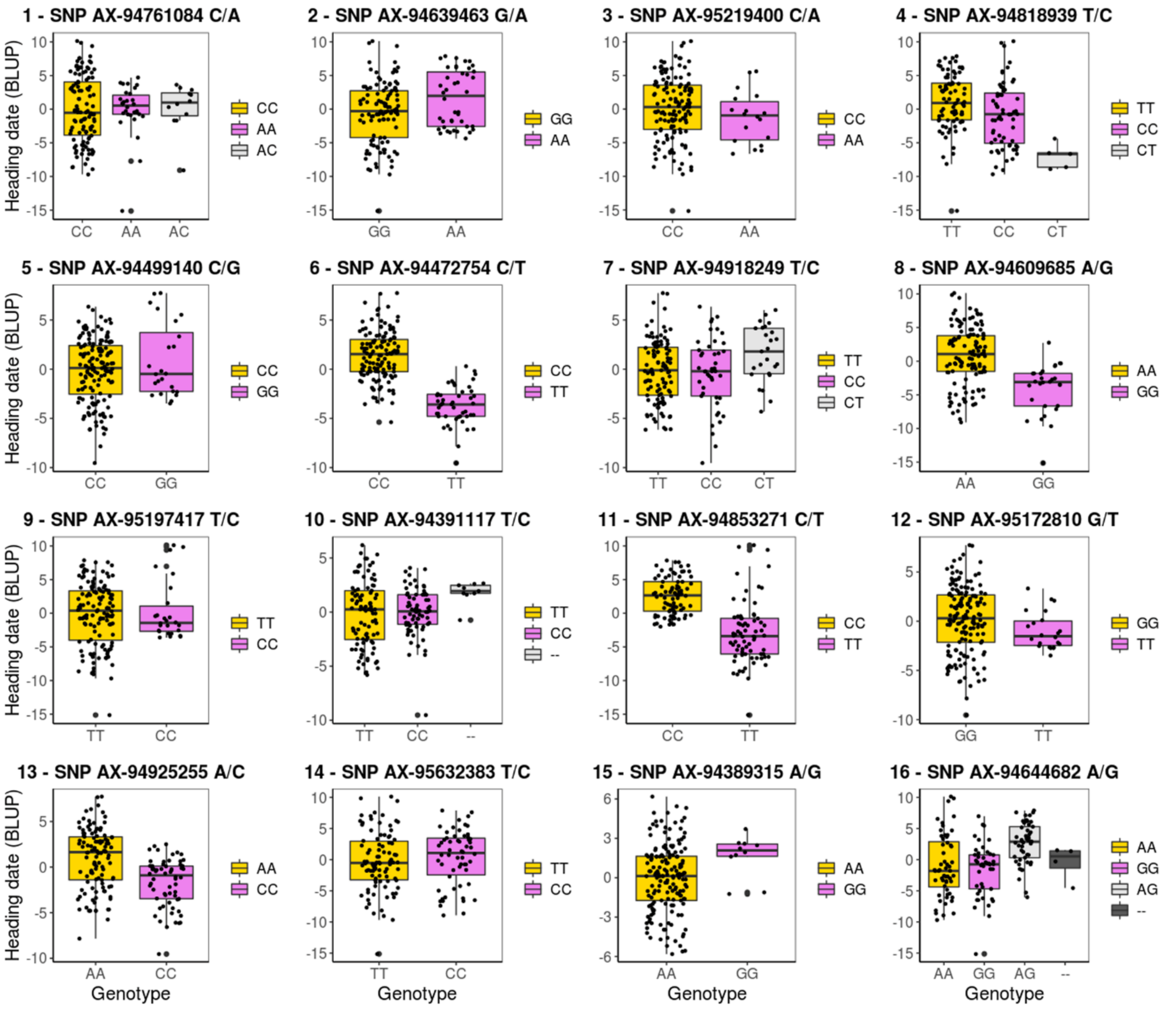

Supplementary Figure 2. Allele-effect box plots for SNP markers with the highest association with heading date. Phenotypical values are in BLUP values used for GWAS. (1) marker AX-94761084 on chromosome (chr) 1B, (2) AX-94639463 on chr 3A, (3) AX-95219400 on chr 3A, (4) AX-94818939 on chr 3B, (5) AX-94499140 on chr 3B, (6) AX-94472754 on chr 4A, (7) AX-94918249 on chr 5A, (8) AX-94609685 on chr 5A, (9) AX-95197417 on chr 5B, (10) AX-94391117 on chr 6A, (11) AX-94853271 on chr 6B, (12) AX-95172810 on chr 7B, (13) AX-94925255 on chr 7B, and (14) AX-95632383 on chr 7B.

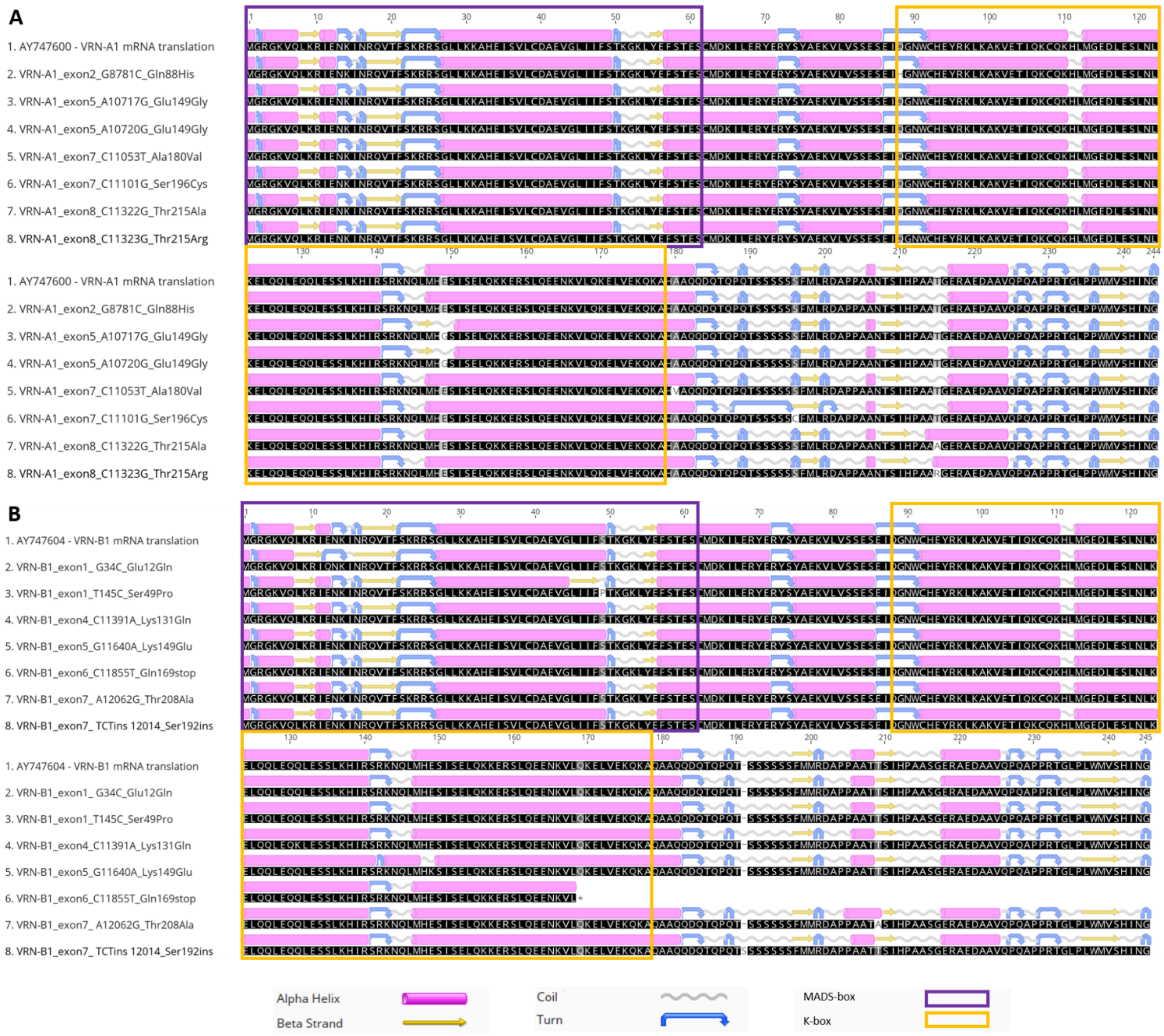

Supplementary Figure 3. Predicted secondary structure of VRN-A1 (A) and VRN-B1 (B) proteins with mutated amino acid sequence (EMBOSS Protein Analysis version 1.0 in the Geneious Prime® 2022.0.1.).

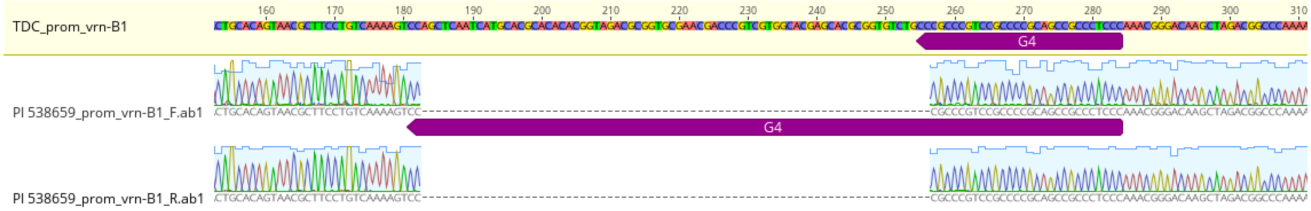

Supplementary Figure 4. Deletion of 74 bp in the *VRN-B1* promoter and reconstitution of G4 motif.

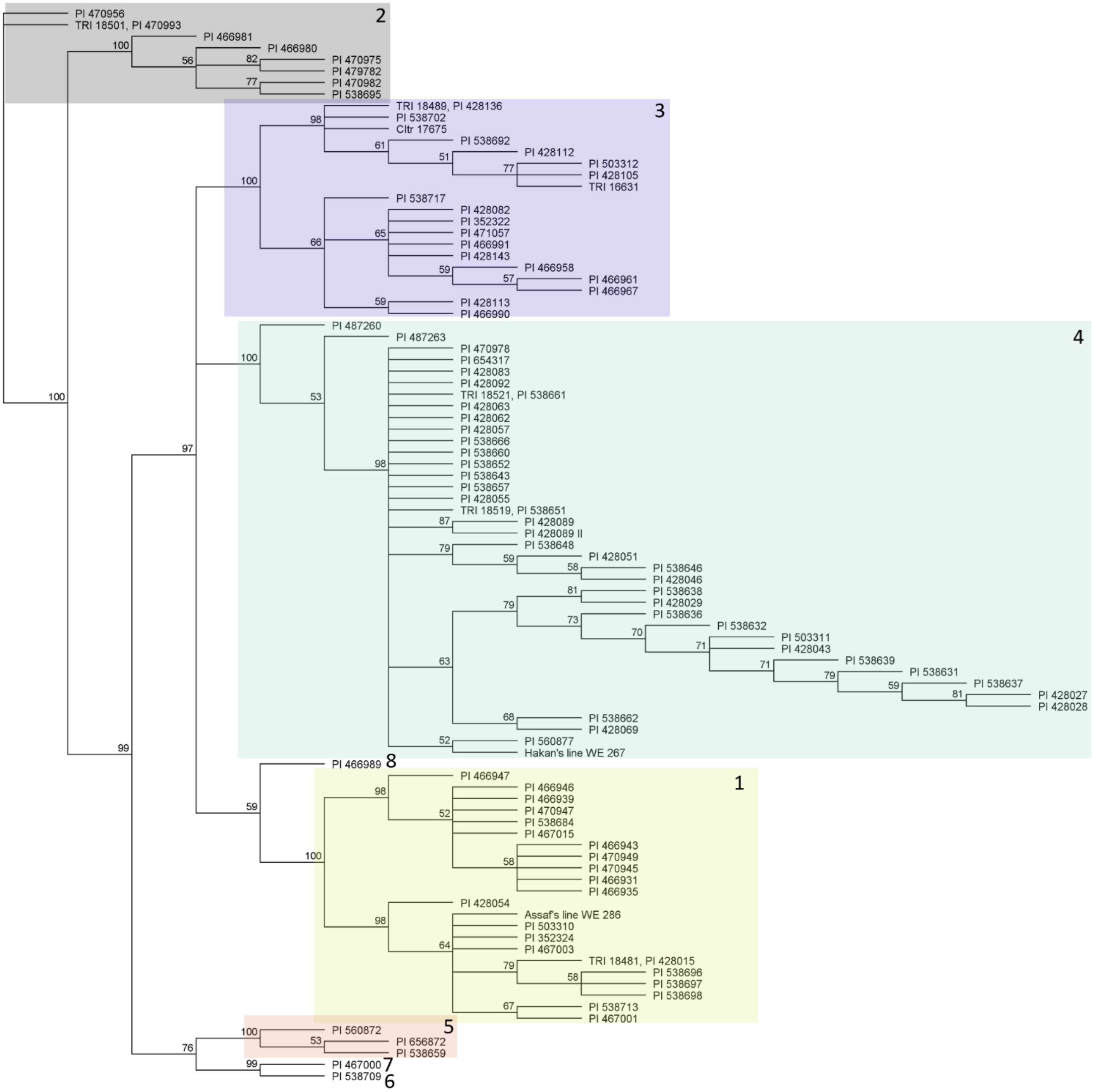

Supplementary Figure 5. Neighbor-Joining consensus tree of *VRN-A1* sequences with eight highlighted groups used for mapping of the *VRN-A1* alleles distribution.

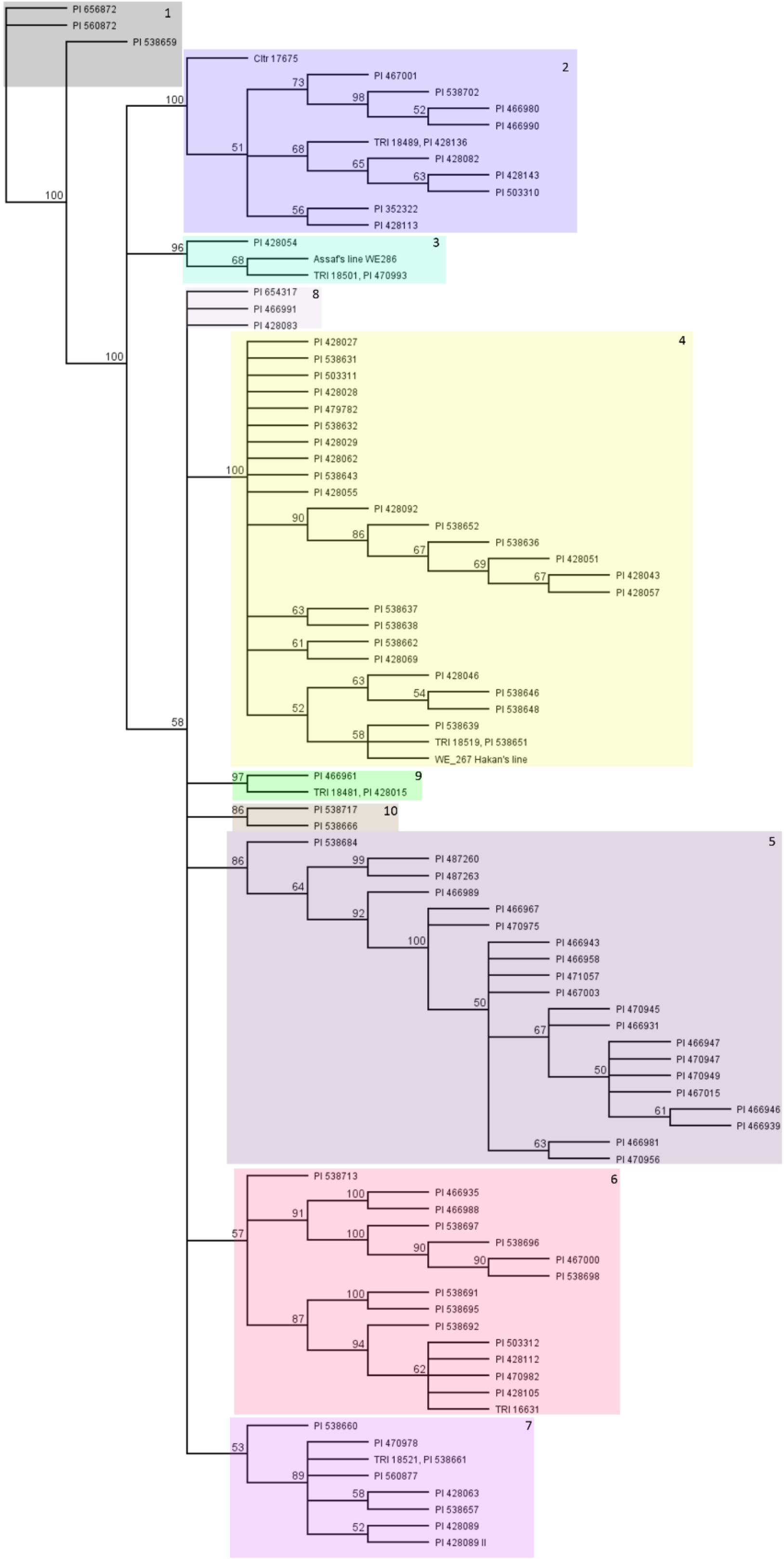

Supplementary Figure 6. Neighbor-Joining consensus tree of *VRN-B1* sequences with ten highlighted groups used for mapping of the *VRN-B1* alleles distribution.
